# The Cell Polarity Protein Scribble Is Involved in Maintaining the Structure of Neuromuscular Junctions, the Expression of Myosin Heavy Chain Genes, and Endocytic Recycling in Adult Skeletal Muscle Fibers

**DOI:** 10.3390/cells14242005

**Published:** 2025-12-16

**Authors:** Lea Gessler, Yongzhi Jian, Nam Anh Ngo, Said Hashemolhosseini

**Affiliations:** 1 Institute of Biochemistry, Medical Faculty, Friedrich-Alexander-University of Erlangen-Nürnberg, Fahrstraße 17, 91054 Erlangen, Germany; lea.gessler@fau.de (L.G.); yzjaikeyan@outlook.com (Y.J.); nam.anh.ngo@fau.de (N.A.N.); 2 Department of Orthopedics, The Fourth Affiliated Hospital of School of Medicine, and International School of Medicine, International Institutes of Medicine, Zhejiang University, Yiwu 322000, China

**Keywords:** Scrib, Scribble, LAP, Erbin, Lano, skeletal muscle, neuromuscular junction

## Abstract

The role of LAP proteins expressed in skeletal muscles (ERBIN, LANO, and SCRIBBLE) and at neuromuscular junctions (NMJs) remains largely unknown. Our previous data demonstrate that LAP proteins are differentially expressed in muscle cells, nerve endings, and terminal Schwann cells, though they are all expressed in myofibers and accumulate at NMJs. ERBIN and SCRIBBLE align with acetylcholine receptor clusters (CHRNs) at the NMJ. In vivo ablation of *Erbin* is associated with smaller CHRN and upregulation of *Lano* and *Scribble*. However, SCRIBBLE was also shown to influence the fate decision of muscle stem cells. Here, we investigated how the absence of SCRIBBLE in skeletal muscle cells might impair skeletal muscle fibers or NMJs. Although conditional *Scribble* knockout mice did not exhibit changes in weight or viability, force per weight decreased slightly. This was supported by compromised neuromuscular transmission and increased NMJ fragmentation. Moreover, *Scribble* knockout muscles transcribe less myosin heavy chain genes. Here, we also showed that RAB5, an effector of endocytic recycling, interacts with all LAP proteins, but in *Scribble* knockout muscles, reduced interaction was detected with ERBIN and LANO. These data suggest that a delicate signaling network employing LAP proteins is necessary for skeletal muscle fibers and NMJs.

## 1. Introduction

LAP proteins constitute a small family of LRR (leucine-rich repeat) and PDZ domain (LAP) proteins, which contain 16 LRRs at their amino-terminus and either none, one, or four PDZ domains at their carboxy-terminus [1,2]. LAPs act as adaptors in different signaling pathways within cells [3,4]. All four reported LAP family members (DENSIN-180, ERBIN, LANO, SCRIBBLE) are expressed in mammals, orthologues of LANO and SCRIBBLE are also expressed in Drosophila and Caenorhabditis [2,5,6,7,8]. LAP proteins frequently accumulate in defined intracellular domains. In polarized epithelial cells—with their distinct apical and basolateral membrane regions—the LAP family members ERBIN, LANO, and SCRIBBLE are enriched at the basolateral surface, where they participate in pathways that control protein trafficking and maintain polarized membrane organization [2,6,7]. Cell polarity is orchestrated by three evolutionarily conserved protein assemblies, the basolateral Scribble module, the Crumbs complex, and the apical PAR complex [9]. The Scribble complex itself consists of the proteins Disks large (Dlg1–4), Lethal giant larvae (Lgl1/2), and SCRIBBLE, all of which reside mainly in the basolateral membrane domain. Beyond this canonical role in polarity, SCRIBBLE has been recognized as a multifunctional scaffold involved in regulating diverse cellular processes, including migration, adhesion, apoptosis, and proliferation. LANO, the only LAP family member lacking a PDZ domain, has been shown to interact with MAGUK proteins in epithelial cells. ERBIN—also referred to as ERBB2IP—is known for binding to the receptor tyrosine kinase ERBB2 via its PDZ domain [2,10]. In neurons, ERBIN and DENSIN-180 are prominent components of the postsynaptic density [10,11]. Within the central nervous system, ERBIN is required for proper remyelination after injury [12], and SCRIBBLE similarly contributes to both myelination and remyelination processes [13].

Myogenesis is divided into distinct phases: embryonic, perinatal, and adult [14]. In mature muscles, progenitors enter quiescence and reside there as muscle stem cells, called satellite cells. *Pax7* is expressed by quiescent satellite cells in adult muscle. Activated satellite cells proliferate after upregulating the myogenic regulatory factor *Myod* (MyoD). After population growth, the majority of satellite cells express *Myog* (Myogenin), maintain MYOD, downregulate *Pax7*, go through myogenic differentiation, and eventually fuse to multinucleated myofibers that express both slow and fast myosin heavy chains. When mature muscle is damaged, satellite cells expand mitotically and differentiate to repair the tissue and re-establish homeostasis [14]. Given that cell-polarity proteins have also been suggested to function as possible regulators of asymmetric cell division, enabling a stem cell to produce a daughter cell that differentiates and another that self-renews, it was questioned whether SCRIBBLE, a polarity and scaffold protein, influences the fate choice of satellite cells during myogenic progression. It was reported that SCRIBBLE is undetectable in quiescent cells but becomes expressed during activation [15]. Furthermore, it was demonstrated that low *Scribble* expression is linked to the proliferative state and the inhibition of self-renewal. Conversely, high SCRIBBLE levels were found to reduce satellite cell proliferation. Ablation of *Scribble* resulted in severe defects in muscle regeneration [15].

Mammalian neuromuscular junctions (NMJs) are small and simple, which makes studying the principles of synapse formation, maintenance, and function easy [16]. After the neurotransmitter acetylcholine binds to nicotinic acetylcholine receptors (CHRNs), cations can pass through the sarcolemma, ensuring proper muscle contraction [16]. Toward the end of embryonic development and around mid-gestation, motoneuron nerve terminals approach the surface of muscle fibers. They stabilize existing CHRN and support their growth by releasing nerve-derived variants of agrin (AGRN), a large heparan sulfate proteoglycan. The receptor of AGRN is composed of LRP4 (Low-Density-Lipoprotein Receptor-Related Protein 4) and the muscle-specific receptor tyrosine kinase MUSK (MuSK) [17,18,19,20,21]. AGRN/LRP4/MUSK signaling ensures proper expression of synaptic genes and clustering of CHRNs, the latter serving as a hallmark for NMJ formation and being detectable by staining with fluorophore-coupled bungarotoxin (BTX). The recruitment of recycled CHRNs to the NMJ requires myosin V motor proteins and regulatory pathways, involving PKA, PKC, CaMKII, and RAB family proteins.

These RAB small GTPases are found at the cytosolic face of specific intracellular membranes, where they regulate distinct steps in membrane trafficking pathways. In their GTP-bound state, RABs recruit specific sets of effector proteins to the membrane. Through these effectors, the RABs regulate the formation of vesicles, the movement of vesicles via actin and tubulin, and membrane fusion. Early endosomes serve as key sorting platforms, directing internalized cargo toward various intracellular destinations [22,23]. RAB5 acts as a central regulator of these organelles and influences nearly all aspects of early endosome dynamics through its extensive network of effector proteins [23,24,25,26]. RAB10, which partially colocalizes with RAB5 on endosomal membranes, contributes primarily to the recycling route that returns cargo to the plasma membrane [27,28]. Members of the LAP protein family—specifically SCRIBBLE and ERBIN—have been implicated in endocytic transport processes [29,30]. In *Caenorhabditis elegans*, ERBIN operates as a RAB5 effector and promotes the activation of RAB10 during recycling transport [29]. SCRIBBLE, by contrast, plays a role in the endocytic sorting of NMDARs through selective interactions with the AP2 adaptor complex [30]. ERBIN has also been shown to regulate TARP γ-2 abundance and is thereby required for proper AMPAR surface expression in interneurons [4].

In mature skeletal muscle, *Erbin*, *Lano*, and *Scribble* are all expressed within muscle fibers and further enriched at neuromuscular junctions (NMJs). These proteins are also present in cultured myogenic cells, with *Lano* and *Scribble* transcript levels increasing as myoblasts differentiate into myotubes [10,31,32]. Beyond its interaction with ERBB2, ERBIN engages with several additional partners; the segment between its LRR and PDZ domains has been reported to bind postsynaptic density protein 95 [10], EBP50 [33], SMAD3 [34], and MUSK [31]. Notably, neither loss of ERBIN nor substitution of endogenous ERBIN with a mutant form lacking the C-terminal PDZ domain (ERBIN-ΔC) disrupted the localization of LAP proteins at NMJs [32]. Interestingly, ERBINs binding sites for SMAD3 and MUSK partially overlap, and ERBIN, MUSK, or constitutively active variants of ERBB2 and MUSK have all been shown to dampen SMAD3-dependent transcription [31]. Furthermore, siRNA-mediated depletion of ERBIN, LANO, or SCRIBBLE in cultured muscle cells reduced both the density and the formation of CHRN and microclusters. Muscle-specific deletion of *Erbin* in mice leads to compensatory upregulation of *Lano* and *Scribble*, diminished grip strength, increased fatigability, and markedly fragmented NMJs with reduced overall volume [32].

Here, we knocked out the *Scribble* gene in forming myotubes and adult muscle fibers by human skeletal actin (HSA)—Cre recombinase in mice. The *Scribble* knockout mice were viable, but their grip strength was significantly reduced due to impaired neuromuscular transmission and NMJ structure. Unlike conditional *Erbin* knockout mice, *Scribble* knockout muscles did not affect the protein amount of ERBIN, only that of LANO. Additionally, transcript levels of *Myh1* (Myosin heavy chain type 2x) and *Myh2* (Myosin heavy chain type 2a) were significantly reduced in *Scribble* knockout muscles. Finally, an interaction was detected between all LAP proteins and RAB5, a protein involved in endocytic recycling. These data point to an important role of SCRIBBLE in skeletal muscle fibers and at NMJs.

## 2. Materials and Methods

### 2.1. Mouse Procedures and Genotyping

All mouse experiments were performed in compliance with national and institutional animal welfare regulations and were approved by the relevant local authorities (animal protection officer, Sachgebiet Tierschutzangelegenheiten, FAU Erlangen–Nürnberg, approvals AZ: I/39/EE006 [Aug 2024], TS-01/2021 [Apr 2021], and RUF-55.2.2-2532-2-1804-24 [Jan 2024]) as well as the regional government (Regierung von Unterfranken). Floxed Scribble mice were generously provided by Dr. Patrick Humbert (La Trobe University, Melbourne, Australia) [35], and HSA-Cre reporter mice have been described previously [36]. Breeding and genotyping procedures followed established protocols [37]. Experimental cohorts consisted of Scribble^loxP/loxP^::HSA-Cre and Scribble^+/loxP^::HSA-Cre littermates aged 3–6 months. Within this age range, and across both sexes, no systematic phenotypic differences were detected; therefore, data were pooled by genotype for all analyses. Additional methodological details are included in the Figure legends. Mice were housed under controlled environmental conditions (22 ± 1 °C; 50–60% relative humidity; 12 h light/dark cycle) with ad libitum access to food and water. Muscle strength was assessed using a Grip Strength Test Meter (Bioseb Vitrolles, France) as previously described [38].

### 2.2. RNA Extraction, Reverse Transcription, PCR

Total RNA was isolated from adult EDL muscle (3–6 months old) using TRIzol reagent (Thermo Fisher Scientific, Dreieich, Germany, 15596026). cDNA was synthesized with M-MuLV Reverse Transcriptase (New England Biolabs; Frankfurt am Main, Germany, M0253) following the manufacturer’s protocol. Quantitative PCR is performed using PowerUp SYBR Green Master Mix (Thermo Fisher Scientific, A25743) and 0.25 µM of each mouse-specific primer (see Supplementary Table S1). All reactions were run in technical triplicate. Amplification was carried out on a C1000 Thermal Cycler coupled to a CFX96 Real-Time PCR Detection System (Bio-Rad, Feldkirchen, Germany) according to the manufacturers’ guidelines. Product sizes were verified by agarose gel electrophoresis. Ct values for each target gene were normalized to the housekeeping gene *Rpl8*, and relative expression levels were calculated using the 2^−ΔΔCT^ method [39,40].

### 2.3. SDS-PAGE, Western Blot, Immunoprecipitation

For SDS-PAGE and immunoblotting, the GAS muscles were homogenized in a lysis buffer containing 50 mM Tris-HCl (pH 8.0), 200 mM NaCl, 0.3% NP-40, 50 mM NaF, 1 mM DTT, 1 mM Na_3_VO_4_, 10 µg/mL aprotinin, 10 µg/mL leupeptin, and 1 mM PMSF [41]. Skeletal muscle homogenates were sonicated for 5 s and centrifuged at 16,100× *g* at 8 °C for 10 min. Cleared lysate was used for immunoblotting experiments. Aliquots of muscle lysates were solubilized in Laemmli buffer (150 mM Tris, pH 6.8, 6% SDS, 30% glycerol, 0.3% bromophenol blue, 3% ß-mercaptoethanol), boiled at 95 °C, and loaded on 8% or 10% SDS-PAGE.

Electrophoretic migration was conducted at 120V for approximately 4 h. Protein transfer to nitrocellulose membranes was carried out for 2 h at 2.5A using a Western-Blot apparatus (Bio-Rad, Feldkirchen, Germany). Membranes were blocked in 5% nonfat dry milk in PBS, 0.1% Tween20 for 1 h at room temperature. Immunoprecipitation was performed with specific antibodies using hind limb muscle lysates. Antibodies were incubated with lysates overnight at 4 °C. The next day, protein A Sepharose beads were added, and incubation was continued for another 3 h. Afterwards, the beads were washed 3 times, boiled in Laemmli buffer, and loaded on SDS-PAGE gel. Primary antibodies were incubated at a 1:1000 dilution. The following antibodies were used: Rab5A ab66746 (Abcam, Cambridge, UK); Scribble sc-55543 (C6), Erbin sc-30054 (H210), Lano sc-243330, GAPDH sc-25778 (SantaCruz, Heidelberg, Germany); Scribble #4475 (Cell Signaling, Frankfurt am Main, Germany). Corresponding secondary antibodies conjugated with horseradish peroxidase (Cell Signaling, 1:1000) were used for 2 h at room temperature. Protein bands were detected by a homemade chemiluminescence reagent composed of 50 mg Luminol (Sigma Aldrich, Taufkirchen, Germany) in 200 mL 0.1 M Tris, pH 8.6, combined with 11 mg para-hydroxy-cumarinic acid (Sigma Aldrich) in 10 mL DMSO. Western blot results were quantified by densitometric analysis using the Fiji image processing package [42]. X-ray films were scanned with an Epson Expression 1600 Pro Scanner (Epson Deutschland GmbH, Düsseldorf, Germany) at 600 dpi. After background subtraction was performed, protein bands of interest were labeled and measured. For quantification, the protein band intensity was normalized to the intensity of the GAPDH protein band of the respective sample [38].

### 2.4. Histochemical Staining, Immunofluorescence Staining, Quantitative 3D Morphometrical Imaging, Color Deconvolution, Fluorescence Microscopy

GAS and SOL muscles from adult control and mutant mice were flash-frozen in 2-methylbutane cooled with liquid nitrogen for histochemical and immunofluorescence analyses. Muscles were sectioned transversely at 10 μm using a cryotome. For hematoxylin and eosin (H and E) staining, sections were incubated for 15 min in Mayer’s hemalum solution (Merck, Darmstadt, Germany, 109249), rinsed for 10 min in tap water, briefly dipped six times in 96% ethanol containing 4% HCl, rinsed again in tap water for 10 min, dehydrated in 70% ethanol for 1 min, stained with eosin (Merck, 115935) for 2 min, dehydrated in 100% ethanol for 1 min, and mounted in DPX, as previously described. For COX staining, sections were incubated at 37 °C for 60 min in 50 mM phosphate buffer (pH 7.4) containing 3,3′-diaminobenzidine tetrahydrochloride (DAB; Sigma-Aldrich, Taufkirchen, Germany, D8001), catalase (20 mg/mL; Sigma-Aldrich, S41168), sucrose, and cytochrome c (Sigma-Aldrich, C2037). Sections were subsequently rinsed in water and mounted in DPX. For immunofluorescence, sections were fixed in 2% paraformaldehyde (PFA), permeabilized for 15 min in 0.3% Triton X-100 with 100 mM glycine, and blocked for 1 h with M.O.M. blocking reagent (Vector Laboratories, Newark, USA). Primary antibodies used included anti-myosin heavy chain type I (BA-F8, DSHB; 1:1000), anti-myosin heavy chain type IIa (SC-71, DSHB; 1:1,000), anti-Scribble (Cell Signaling, Frankfurt am Main, Germany; 1:1000), anti-Lano (T14, Santa Cruz, Heidelberg, Germany, 1:1000), and anti-Erbin (H210, Santa Cruz; 1:1,000). Detection was achieved with Alexa Fluor 488- and 546-conjugated secondary antibodies (Thermo Fisher Scientific, Dreieich, Germany; 1:1000). All stained sections were imaged using a Zeiss Axio Examiner Z1 microscope equipped with an AxioCam MRm camera and ZEN Blue software (release 3.6, Carl Zeiss MicroImaging, ZEISS, Oberkochen, Germany). Images were processed and analyzed using Fiji (vers. 1.54p) and ZEN Blue (Vers. 3.6.095.02000). For quantitative 3D morphometric analysis, SOL muscles were dissected and fixed in 4% PFA for 1 h at 4 °C. Muscle bundles containing 5–10 fibers were stained with α-bungarotoxin (BTX; Thermo Fisher Scientific, Dreieich, Germany, 1:2500) for 1 h at room temperature, washed three times in PBS (the penultimate wash included DAPI, 1:10,000), and mounted in Mowiol. Confocal images of NMJs were acquired with a 63× oil objective (Zeiss Examiner Z1, ZEISS, Oberkochen, Germany) at 250 ms exposure. After deconvolution, NMJ volume, surface area, and number of fragments were quantified using ZEN Blue (release 3.6). Over 50 NMJs per genotype were analyzed [43]. COX staining intensity was quantified using the Color Deconvolution 2 plugin in Fiji [44,45].

### 2.5. Nerve Muscle Preparation and Electrophysiological Recordings

Diaphragm–phrenic nerve preparations were maintained ex vivo in Liley’s solution equilibrated with 95% O_2_ and 5% CO_2_ at room temperature [46]. The recording chamber (≈1 mL) was continuously perfused at 1 mL/min. The phrenic nerve was drawn into a suction electrode and stimulated with 0.1 ms pulses [4]. The preparation was placed on the stage of a Zeiss Axio Examiner Z1 microscope (Carl Zeiss MicroImaging) fitted with incident light fluorescence illumination with filters for 547 nm/red (Zeiss filter set 20) fluorescing fluorophore. First, a micropipette with a tip diameter of approximately 10 µm was used to record the compound muscle action potential (CMAP) in a bathing solution. The electrode was positioned to minimize the latency of the major negative peak. Then, the electrode was positioned 100 µm above the muscle surface, and the CMAP was recorded. For recordings in the presence of d-tubocurarine, the chamber was filled with 2 mL of d-tubocurarine chloride (500, 800, or 1000 nM) (Sigma Aldrich). During curare treatment, 25 repetitive nerve stimulations (5 Hz) were performed every 2 min, and the ratio of CMAP amplitudes (mean of the 20th–25th/2nd) was calculated [38,43,47].

For recording endplate potentials (EPPs) and endplate currents (EPCs), µ-conotoxin GIIIB (µ-CTX, 2 µM; Peptide Institute, Osaka, Japan) was added to Liley’s solution to block muscle action potentials [48,49]. EPPs were evoked at 5 Hz for 5 s and 20 Hz for 10 s, and their decrement was calculated using the mean of the first and last five responses. Clustered CHRNs at the neuromuscular junction (NMJ) were labeled simultaneously by adding 0.5 × 10^−8^ M α-bungarotoxin (BTX; Life Technologies) to the solution. In some cases, the effect of the toxin wore off after 1–2 h, and contractions resumed in response to nerve stimulation; these preparations were re-exposed to µ-CTX.

Two intracellular electrodes (10–15 MΩ) were positioned within 50 µm of the NMJ under visual guidance [32]. One electrode delivered current to maintain the membrane potential at −75 mV ± 2 mV, while the second recorded voltage transients. Signals were amplified using an Axoclamp 900A and digitized at 40 kHz with a Digidata 1440A controlled by pCLAMP 10 (Molecular Devices, San Jose, USA). Voltage signals were filtered at 3 kHz, and currents at 1 kHz, using an 8-pole Bessel filter. EPCs were recorded using the two-electrode voltage-clamp configuration of the Axoclamp 900A, with clamp gains set between 300 and 1000 to reduce voltage transients to <3% of their unclamped amplitudes.

At each NMJ, 50–100 spontaneous quantal events were collected over 1 min. Records were analyzed in pCLAMP vers. 10.3; spontaneous events were detected using the “template search” tool and manually curated to remove artifacts. Amplitude and frequency were determined by averaging all events recorded from each NMJ [43].

### 2.6. Statistical Analysis

Statistical analyses and graph generation were performed using GraphPad Prism 10 (GraphPad Software, San Diego, USA). Outliers were identified by Prism and excluded from the dataset and corresponding graphs. Unless otherwise specified, comparisons between two groups were made using an unpaired Student’s *t*-test. Graphs display individual data points with the mean ± SD. *p*-values are reported in GraphPad style, showing four digits after the decimal point with a leading zero: ns (not significant) *p* > 0.05, * *p* ≤ 0.05, ** *p* ≤ 0.01, *** *p* ≤ 0.001, **** *p* ≤ 0.0001.

## 3. Results

### 3.1. Muscle Grip Strength Is Reduced in Conditional Muscle-Specific Scribble Knockout Mice

**Figure 1:**
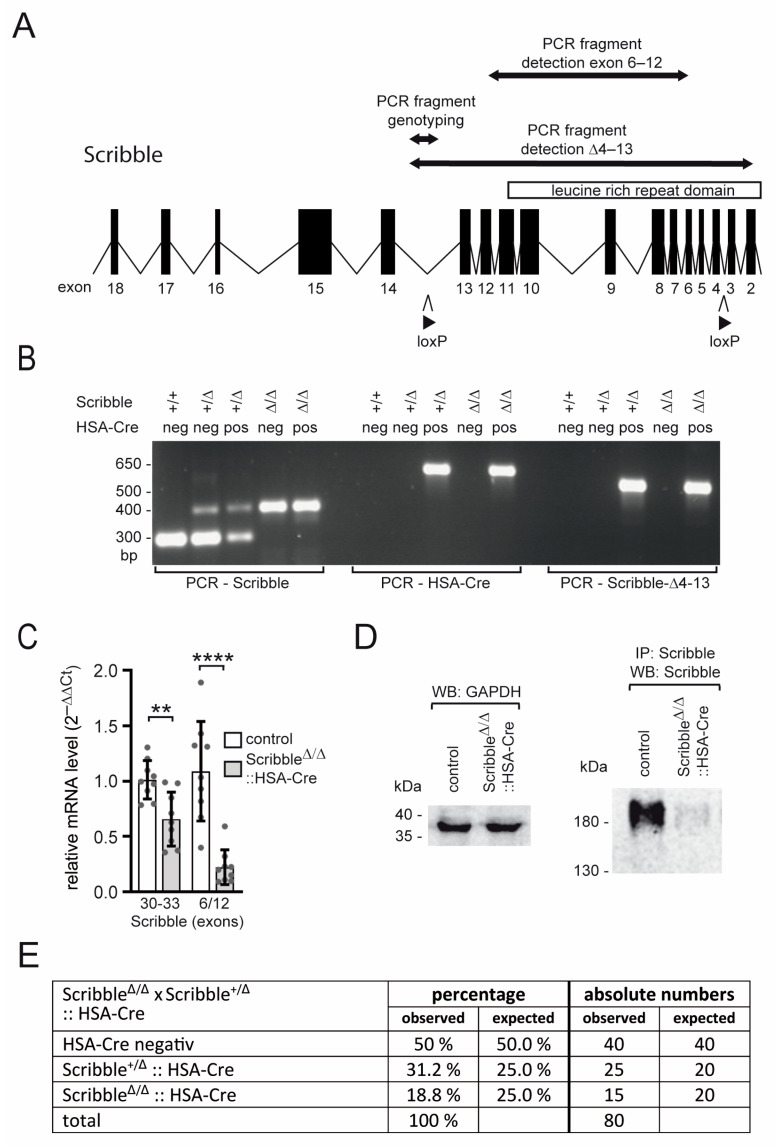
Generation and analysis of conditional muscle-specific *Scribble*-deficient mice. (**A**) Schematic representation of the wildtype *Scribble* allele from exon 2 to 18. Insertion sites of loxP sequences, exons representing part of the targeting construct, and PCR primers used to amplify *Scribble* DNA fragments are depicted. Excision of the loxP-flanked exons 4 to 13 was accomplished by crossing floxed *Scribble* mice to human skeletal actin (HSA)-Cre mice. (**B**) Images show PCR fragments obtained using different primer pairs (see (**A**)) to detect floxed *Scribble* alleles (left), HSA-Cre (middle), or *Scribble* allele with deletion of exons 4 to 13, Δ4 to 13 (right). (**C**) The graph summarizes PCR amplification with different primer pairs detecting either *Scribble* mRNA part, exon 30 to 33, or exon 6 to 12. The robust decrease in Δ6 to 12 mRNA levels reflects excision of loxP-flanked exons in floxed *Scribble* mice. One EDL muscle from each mouse (minimum three mice per genotype, ~4 months of age) was analyzed. PCR was performed three to five times in triplicate. (**D**) Images of Western blot membranes show the presence of GAPDH in wild-type and conditional *Scribble*-deficient mice, but SCRIBBLE only in wild-type and not in *Scribble* knockout muscles. One gastrocnemius muscle from each mouse (minimum five mice per genotype, 4 months of age) was analyzed. (**E**) The Table summarizes the genotypes of *Scribble* offspring, demonstrating their Mendelian distribution. *p* > 0.05, ** *p* ≤ 0.01, **** *p* ≤ 0.0001.

**Figure 2:**
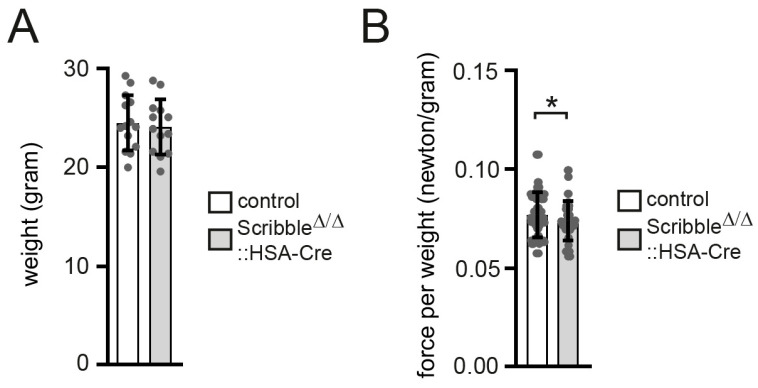
Decreased muscle strength in conditional muscle-specific *Scribble*-deficient mice. (**A**) The body weight of control and muscle-specific *Scribble*-deficient mice is summarized in a graph. Weight measurements were taken once per mouse (minimum 13 mice per genotype, 3–6 months of age). (**B**) The muscle force of control and muscle-specific *Scribble*-deficient mice is calculated per body weight of the same mice and presented as newtons per gram in a graph. Grip strength measurements were taken four to six times (minimum 13 mice per genotype, 3–6 months of age). Note, muscle-specific *Scribble*-deficient mice display mildly, but significantly, less grip strength in comparison with controls. *p* > 0.05, * *p* ≤ 0.05.

Constitutive *Scribble* knockout mice die shortly after birth and exhibit a spectrum of developmental defects, most notably in the nervous system and overall body morphology [50]. In contrast, the influence of SCRIBBLE on tissue organization appears less prominent in several other organ systems; however, the LAP family members ERBIN, LANO, and SCRIBBLE show distinct expression in skeletal muscle both during development and in adulthood [10,15,31,32]. To elucidate the role of SCRIBBLE in myotube formation, mature muscle function, and NMJ organization, we generated skeletal muscle–specific *Scribble* knockout mice (Figure 1). A conditional *Scribble* allele carrying loxP sites flanking exons 4–13 (Figure 1A), previously described in the literature [35], was used for this purpose. These mice were crossed with HSA-Cre transgenic animals, which express Cre recombinase in developing myotubes and mature myofibers. This strategy results in the deletion of most of the coding sequence for the SCRIBBLE LRR domain, effectively abolishing SCRIBBLE function in skeletal muscle [36]. The deletion is confirmed on the DNA, RNA, and protein levels by PCR genotyping and Western blot in the gastrocnemius (GAS) muscle in co-presence of the HSA-Cre recombinase, but not in its absence (Figure 1B–D). Using different *Scribble*-specific primer pairs, we detected different genomic fragments of the *Scribble* locus. One area comprises exons 4 to 13 and is flanked by loxP. Another primer set detected the *Scribble* genomic area between exons 6 and 12. This region is absent in *Scribble* knockout mice. Finally, a primer pair that was also used for genotyping *Scribble* offspring detected a genomic fragment between exons 13 and 14 (Figure 1A,B; labeled PCR *Scribble*). The resulting PCR fragments demonstrated a lack of exons 4 to 13 and a general reduction in *Scribble* mRNA in *Scribble* knockout mice compared to controls. (Figure 1C). Muscle-specific *Scribble* knockout mice are viable and fertile, and the genotypes of their offspring follow the expected Mendelian distribution (Figure 1E), suggesting that there was no developmental lethality. The weight of adult muscle-specific *Scribble* knockout mice is not different from that of control mice (Figure 2A), but the force of mutant mice is slightly and significantly reduced (Figure 2B). No changes were observed when comparing muscle-specific *Scribble* knockout mutant mice with heterozygous *Scribble* knockout mice, providing evidence of a lack of haploinsufficiency.

### 3.2. The Absence of Scribble Did Not Affect ERBIN, Only LANO Protein Levels, but Reduced Interaction of All LAP Proteins with RAB Indicated Involvement in Endocytic Recycling

**Figure 3:**
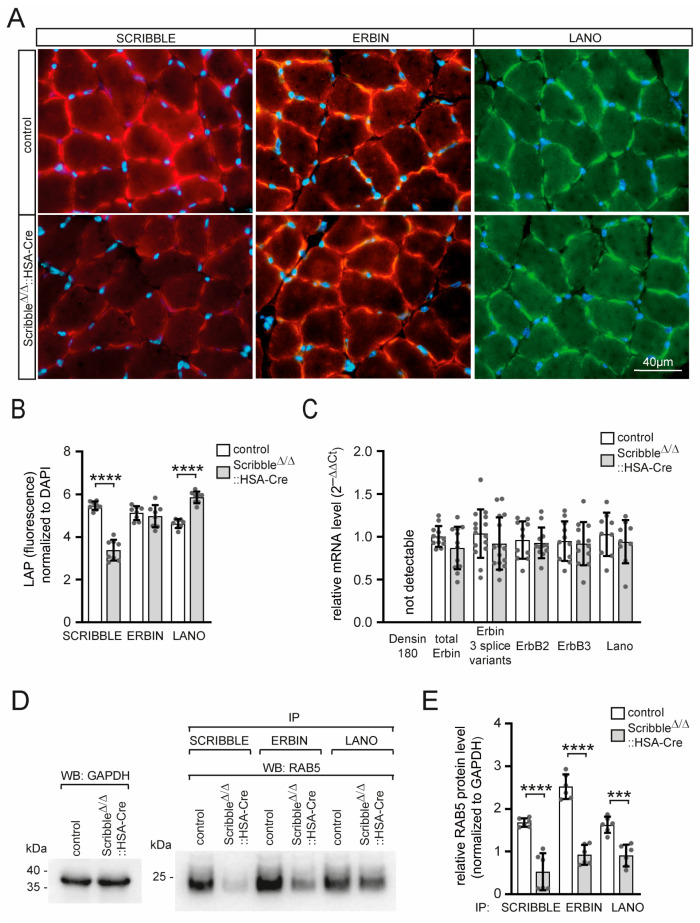
In muscles devoid of SCRIBBLE, diminished levels of ERBIN and LANO interact with RAB5. (**A**) Images show representative hindlimb muscle cross sections of control or muscle-specific *Scribble*-deficient mice after immunofluorescence staining with SCRIBBLE, ERBIN, or LANO-specific antibodies. Note, the secondary antibody is responsible for the background stain in *Scribble*-deficient muscle cross sections. One gastrocnemius/SOL muscle from each mouse (minimum three mice per genotype, ~4 months of age) was analyzed. (**B**) The immunofluorescence intensities detected in (**A**) are presented in a graph. (**C**) The relative transcript levels of several markers are summarized in the graph. Note, *Densin-180* is not upregulated in *Scribble*-deficient muscles, and *Lano, Erbin, and Erbb2/Erbb3* mRNA levels are unchanged. One EDL muscle from each mouse (minimum three mice per genotype, ~4 months of age) was analyzed. PCR was performed three to five times in triplicate. (**D**) Representative Western blot images show RAB5 being co-immunoprecipitated by individual precipitation of SCRIBBLE, ERBIN, or LANO. One gastrocnemius muscle from each mouse (minimum five mice per genotype, 6 months of age) was analyzed. Due to a lack of SCRIBBLE protein, lysates of *Scribble*-deficient muscles do not co-precipitate RAB5. Note, in muscle-specific *Scribble*-deficient mice, significantly less RAB5 protein is co-immunoprecipitated by ERBIN or LANO. (**E**) Protein amounts shown by Western blots (see (**D**)) are quantified and presented in the graph. *p* > 0.05, *** *p* ≤ 0.001, **** *p* ≤ 0.0001.

Previous studies have shown that the levels of *Lano* and *Scribble* are higher in homozygous *Erbin*-null mice than in heterozygous litters [32]. Here, we investigated whether the levels or subcellular localization of ERBIN or LANO change in the absence of SCRIBBLE. Using immunofluorescence staining on muscle cross sections with antibodies specific to one of the three LAP proteins, we detected a significant reduction in SCRIBBLE immunofluorescence staining in mutant mice compared to control mice. Staining levels did not change for ERBIN, but LANO is upregulated (Figure 3A,B). However, transcript levels of *Erbin* and *Lano* are unchanged in mutants compared to controls (Figure 3C). Another LAP protein member, Densin-180, was previously reported not to be expressed in skeletal muscles [31], and *Scribble* knockout mice still lack ectopic expression of *Densin-180* in their skeletal muscles (Figure 3C). Different ERBIN isoforms—full-length and two lacking either exon 22 or exons 22 and 23—were reported to be expressed in skeletal muscle [31]. The expression levels of none of these ERBIN isoforms appeared significantly impaired in *Scribble* knockout muscles, though the *Erbin* transcript levels are slightly reduced (Figure 3C). ERBIN was originally identified as an ERBB2-interacting protein [2]. In this sense, transcript levels are investigated and found to be unchanged for *Erbb2* and *Erbb3* (Figure 3C). Furthermore, the LAP protein homolog of SCRIBBLE/ERBIN, LET-413, was reported to act as a RAB5 effector during endocytic recycling in Caenorhabditis elegans [29]. To determine whether mammalian LAP proteins interact with RAB GTPases, which are known to mediate endocytic recycling, in skeletal muscle, we immunoprecipitated ERBIN, LANO, and SCRIBBLE to detect co-immunoprecipitated RAB5 protein (Figure 3D,E). RAB5 is indeed co-immunoprecipitated by each of the muscular LAP proteins (Figure 3D,E). Notably, no RAB5 was co-immunoprecipitated by SCRIBBLE immunoprecipitation in *Scribble* knockout muscle lysates due to the absence of SCRIBBLE (Figure 3D). Interestingly, the amount of RAB5 protein interacting with ERBIN or LANO was significantly reduced in *Scribble* knockout mice compared to control mice (Figure 3D,E). Overall, these findings suggest an exciting, previously unknown, involvement of LAP proteins in endocytic recycling in mice.

### 3.3. Skeletal Muscle in Scribble Mutant Mice Does Not Show Any Changes in General Muscle Histology or Energy Metabolism

**Figure 4:**
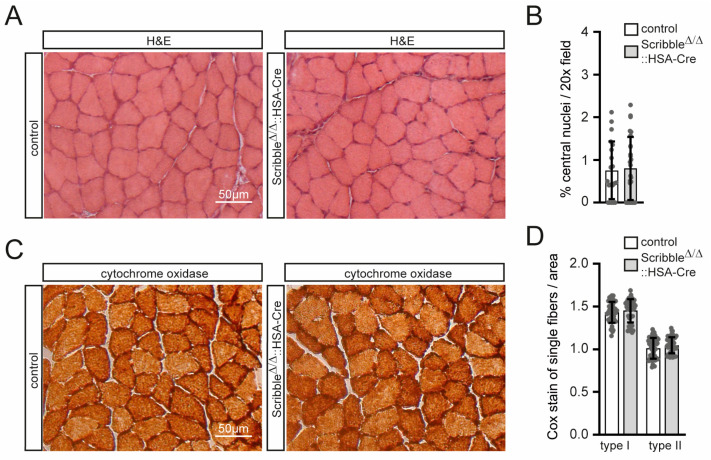
Skeletal muscle histology is not altered in muscles that lack SCRIBBLE. (**A**) Images show representative hindlimb muscle cross sections of control or muscle-specific *Scribble*-deficient mice stained by hematoxylin and eosin. (**B**) The graph shows the unchanged number of central nuclei in cross-sectioned hindlimb SOL muscle of control in comparison with muscle-specific *Scribble*-deficient mice. (**C**) Hindlimb cross sections of control or muscle-specific *Scribble*-deficient mice stained by cytochrome oxidase (COX) staining do not apparently look different. (**D**) By quantitative evaluation of COX staining intensities upon color deconvolution, still no alterations were detected. One SOL muscle from each mouse (minimum three mice per genotype, 4–5 months of age) was analyzed.

To achieve an initial idea of muscle impairments in *Scribble* knockout mice, cross sections of their GAS and soleus (SOL) hind limb muscles were examined for histological changes using typical hematoxylin and eosin staining (data are shown for SOL, Figure 4A). No apparent differences are detected between mutant and control mice (Figure 4A). Additionally, we did not observe any signs of myopathy in individual mutant mice because the number of fibers with centrally located nuclei or apoptotic cells was similar in both genotypes (Figure 4B). Next, we performed cytochrome oxidase (COX) histochemical staining on muscle cross sections. This assay highlights fibers with elevated mitochondrial content—such as slow type I fibers and fast oxidative type IIa fibers—by producing a darker reaction product. Qualitative comparison of stained hindlimb sections reveals no noticeable differences in COX signal intensity between mutants and control animals (Figure 4C). A more quantitative analysis was performed using Fiji software and color deconvolution, but again, no changes were detected when comparing mutant and wild-type muscle cross sections (Figure 4D).

### 3.4. Myosin Heavy Chain Transcript Levels and Cross-Sectional Areas of Skeletal Muscle Fibers Are Altered in Conditional Scribble-Deficient Mice

**Figure 5:**
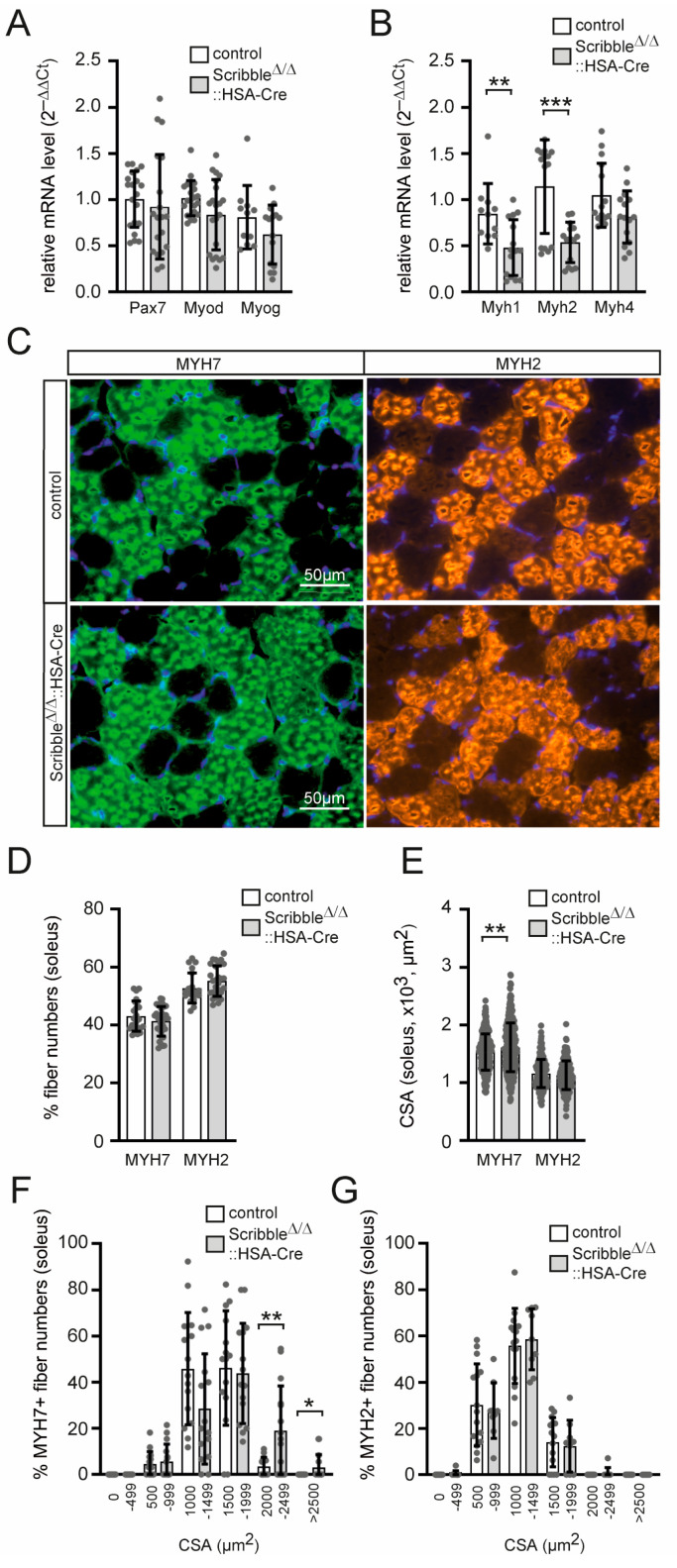
Skeletal muscles lacking SCRIBBLE exhibit a diminished level of transcription of the myosin heavy chain genes 1 and 2. (**A**) The graph summarizes the comparative analysis of transcript levels of typical myogenic markers, *Pax7*, *Myod*, and *Myog* of control and *Scribble*-deficient muscles. (**B**) The transcript level analysis of myosin heavy chain genes in EDL muscle revealed significantly diminished transcript levels for *Myh1* and *Myh2*. One EDL muscle from each mouse (minimum three mice per genotype, ~4 months of age) was analyzed. PCR was performed three to five times in triplicate. (**C**) Images of typical myosin heavy chain immunostains for slow (type 1, MYH7) and fast (type 2, MYH2) cross-sectioned SOL fibers (minimum three mice per genotype, 4–5 months of age). (**D**) The graph shows quantification of MYH7 or MYH2 SOL fibers numbers in either the presence or absence of *Scribble*. (**E**) Cross-sectional areas (CSA) of fibers (as in D) were analyzed for control or muscle-specific Scribble-deficient mice and are presented in a graph. (**F**,**G**) CSA frequency distribution was calculated by plotting % of MYH-positive fibers against CSA size ranges. Note, there are more of the larger fibers within the MYH7-positive fibers. *p* > 0.05, * *p* ≤ 0.05, ** *p* ≤ 0.01, *** *p* ≤ 0.001.

SCRIBBLE has been reported to be strongly up-regulated during the differentiation of myoblasts into myotubes, and it has been identified as a regulator of myogenic progression [15,31]. In *Scribble* knockout muscles, no difference in transcript levels is observed for *Pax7*, *Myod*, or *Myog* (Figure 5A). Surprisingly, late differentiation markers such as the myosin heavy chain (*Myh*) genes are differently regulated in *Scribble* knockout muscles compared to controls (Figure 5B). *Myh1* (expressed in type IIx muscle fibers) and *Myh2* (expressed in type IIa muscle fibers) exhibited significantly lower transcript levels, while *Myh4* (expressed in type IIb muscle fibers) exhibited only slight downregulation (Figure 5B). Next, cross sections of hindlimb muscles are immunostained to detect myosin heavy chain slow and fast subtypes MYH7 and MYH2 (Figure 5C). Counting fiber numbers revealed no change in the distribution of fiber types (Figure 5D). Analysis of cross-sectional areas (CSAs) of individual fibers reveals no change in MYH2-positive fibers but a significant increase in CSAs of MYH7-positive fibers (expressed in type I muscle fibers) (Figure 5E). Further detailed inspection of CSA frequency distribution elucidates higher MYH7-positive, but not MYH2-positive, fiber numbers for the fiber size range from >2000 µm^2^ in comparison with controls (Figure 5F,G). Overall, impaired transcription of myosin heavy chain genes may indicate Scribble’s involvement in late steps of muscle cell differentiation.

### 3.5. Neuromuscular Transmission Was Compromised in Skeletal Muscles of Mutant Scribble Mice

**Figure 6:**
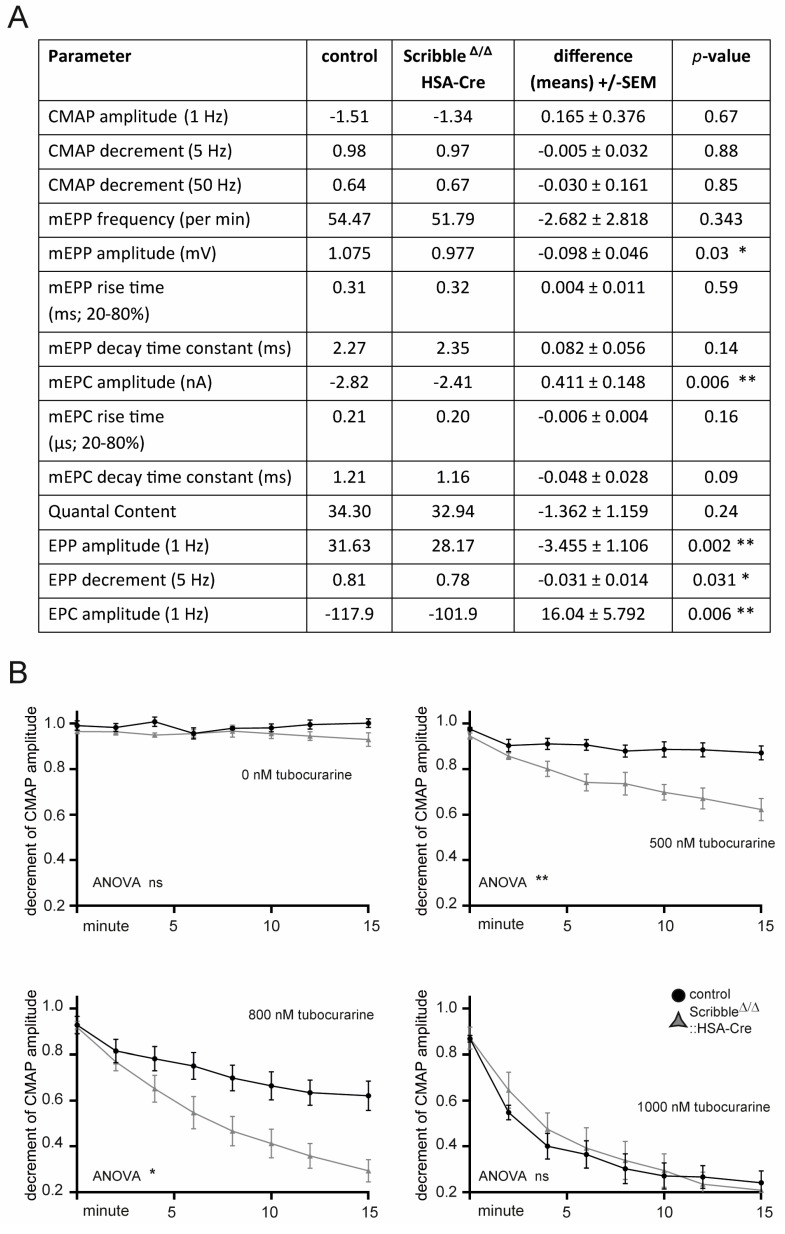
Electrophysiological recordings demonstrated that muscles lacking SCRIBBLE exhibited impaired neuromuscular transmission. (**A**) A number of different parameters were investigated by electrophysiological recordings using diaphragm muscles of mice with indicated genotypes in a nerve-dependent or nerve-independent manner, and are summarized in the depicted Table. Abbreviations: CMAP compound muscle potential, (m)EPP (miniature) End Plate Potential, (m)EPC (miniature) End Plate Current. Single fibers (*n* ≥ 20) of diaphragm muscles (four mice per genotype, 4–6 months of age) were intracellularly recorded. The same diaphragms were recorded extracellularly. (**B**) CMAP measurements in control and *Scribble*-deficient diaphragms under untreated conditions and in the presence of increasing concentrations (500, 800, and 1000 nM) of d-tubocurarine. *Scribble*-deficient diaphragms show a significantly higher decrease in CMAP, which is already displayed at 500 nM and 800 nM d-tubocurarine, thus unmasking a reduced safety factor in the absence of *Scribble*. For 500 nM d-tubocurarine: data point 6 (*p* adj. 0.028), 7 (*p* adj. 0.044), and 8 (*p* adj. 0.027), for 800 nM d-tubocurarine: data point 7 (*p* adj. 0.031) and 8 (*p* adj. 0.017) were significantly decreased in *Scribble* knockout diaphragm muscle. Significance was analyzed by two-way RM ANOVA (Sidak comparison test). Error bars indicate S.E.M. Diaphragm muscle from each mouse (minimum four mice per genotype, 4–6 months of age) was analyzed. ns: not significant; *p* > 0.05, * *p* ≤ 0.05, ** *p* ≤ 0.01.

*Scribble* knockout mice have less grip strength than control litters (Figure 2B). Changed levels of different *Myh* genes (Figure 5B) and CSAs in MYH7-positive fibers (Figure 5E) may explain the decreased grip strength. Alternatively, impaired neuromuscular transmission may also contribute to diminished grip strength, consistent with previous studies showing SCRIBBLE to be subcellularly enriched at NMJs [31,32]. To better understand neuromuscular transmission properties in *Scribble* knockout mice, several different electrophysiological recordings were performed using the diaphragm muscle. Nerve-dependent extracellular recordings of compound muscle action potentials (CMAPs) revealed no alterations when comparing CMAP amplitudes at 1 Hz or CMAP decrements at 5 or 50 Hz between *Scribble* knockout and control mice (Figure 6A). Intracellular recordings employed µ-conotoxin GIIIB (µCTX, 2–3 µM) to block muscle fiber action potentials and prevent contraction, as previously described [32], using 3- to 6-month-old mice. Nerve-independent extracellular recordings of miniature endplate potentials (mEPPs) and currents (mEPCs) revealed slightly, yet significantly, reduced amplitude heights in *Scribble* knockout mice compared to control mice (Figure 6A). Furthermore, nerve-dependent intracellular recordings revealed reduced endplate potential (EPP) and current (EPC) amplitudes at 1 Hz and a reduced EPP decrement at 5 Hz (Figure 6A). The reduced mEPP and EPP amplitudes could be due to either decreased quantal size or reduced postsynaptic sensitivity to individual quanta. However, this does not appear to be the case, as the mean quanta were unchanged in *Scribble* knockout mice (Figure 6A). As previously mentioned, assessing neuromuscular transmission by stimulating the phrenic nerve at 5 Hz resulted in a greater decrement in the diaphragm of *Scribble* knockout mice (Figure 6A). Finally, we assessed the neuromuscular safety factor, defined as the margin by which synaptic depolarization exceeds the threshold required to trigger a muscle action potential [51]. We recorded compound muscle action potentials (CMAPs) from the diaphragms of *Scribble* knockout and control mice while applying increasing concentrations of d-tubocurarine to induce a partial blockade of CHRNs. Under these conditions, *Scribble*-deficient muscles exhibit a pronounced reduction in CMAP amplitude during repetitive stimulation, whereas control muscles show little to no change (Figure 6B). These findings indicate that the safety factor is compromised in the absence of *Scribble* and suggest that the absence of *Scribble* contributes to impairments in neuromuscular synaptic transmission.

### 3.6. The NMJs of Mutant Scribble Knockout Muscles Are Fragmented, and Less Acetylcholine Receptor Alpha Subunit Is Expressed

**Figure 7:**
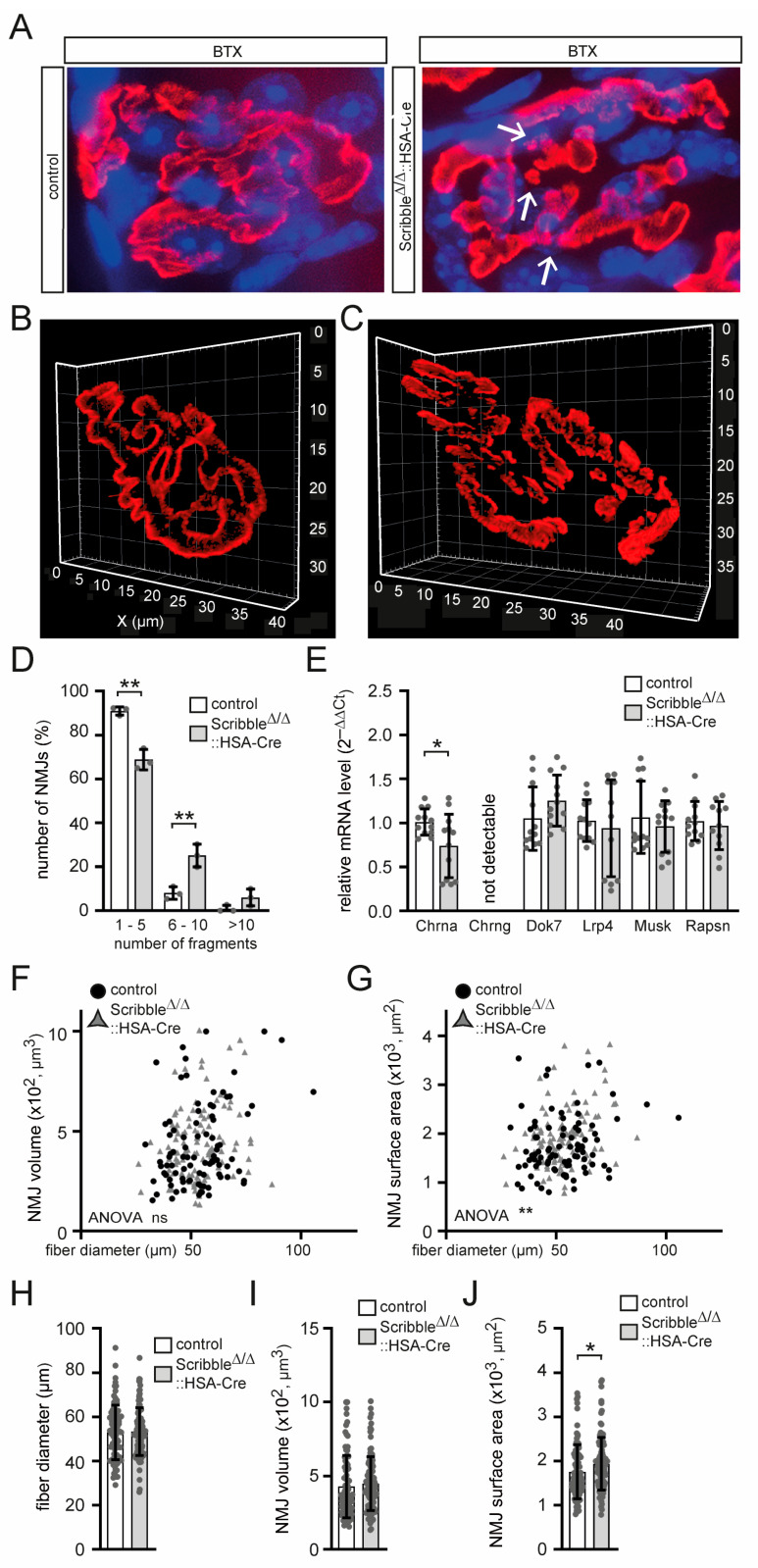
Fluorophore-stained neuromuscular junctions are fragmented in muscles lacking SCRIBBLE. (**A**) 2D images of typical pretzel-shaped NMJs of adult control or *Scribble*-deficient SOL muscle fibers are shown (minimum three mice per genotype, ~4 months of age). Note, NMJs of *Scribble*-deficient mice are of fragmented appearance; the fragmentation sites are marked by arrows. DAPI-stained nuclei appear unchanged. (**B**,**C**) Representative 3D images confirm findings depicted by the 2D images (as shown in (**A**)). (**D**) Quantification of fragmentation grade for control and *Scribble*-deficient muscles is plotted for three groups with different numbers of fragments (1–5, 6–10, >10). (**E**) Late myogenic marker and NMJ transcript levels are analyzed, quantified, and presented in a graph. (**F**,**G**) More than 100 NMJs per genotype were imaged, deconvoluted, and quantified regarding NMJ volume (**F**) or surface area (**G**), plotted individually versus fiber diameter. Note, in accordance with NMJ fragmentation, surface area of NMJs is increased in *Scribble*-deficient SOL muscle fibers. (**H**–**J**) The graphs present a comparison of NMJs of control and *Scribble*-deficient SOL muscle fibers regarding fiber diameter, NMJ volume, and surface area. 30–50 individual NMJs (n ≥ 30–50) of SOL muscles of mice were analyzed. ns: not significant; *p* > 0.05, * *p* ≤ 0.05, ** *p* ≤ 0.01.

After observing impaired neuromuscular transmission in the muscles of *Scribble* knockout (see Figure 6), we questioned whether the morphology of the neuromuscular junctions (NMJs) was altered in these mice compared to control mice. A preliminary examination of a few BTX-stained NMJs revealed fragmentation in the *Scribble* knockout muscles (Figure 7A–D). However, the synaptic nuclei beneath the postsynaptic membrane do not appear different between the control and *Scribble* knockout mice (Figure 7A). The mRNA levels of various NMJ markers reveal lower amounts of CHRNAs in the muscles of the *Scribble* knockout mice (Figure 7E). The mRNA levels of the other analyzed NMJ markers are unremarkable in terms of abundance (Figure 7E). Transcription of *Chrng* is not induced in the mutant muscles, thus ruling out regeneration processes (Figure 7E). More detailed, quantitative analyses of BTX signal intensities linked to NMJs reveal small, yet significant, increases in NMJ volumes and surface areas. These increases are consistent with the observed NMJ fragmentation (Figure 7F–J).

## 4. Discussion

Three of the four LAP protein family members, ERBIN, SCRIBBLE, and LANO, are known to be expressed during skeletal myogenesis, in adult myofibers, and at different cellular entities of neuromuscular junctions [10,15,31,32]. Here, we focus on SCRIBBLE, which is generally known to act as a master scaffold adaptor protein that facilitates key molecular interactions at distinct subcellular localizations [52]. Interestingly, SCRIBBLE appears to have multiple functions in skeletal muscle cells. Its expression increases in committed mononuclear muscle stem cells [15] and in differentiated, multinuclear myotubes [31]. It also accumulates subcellularly at neuromuscular junctions under the postsynaptic apparatus and in nerve terminal endings, but not in terminal Schwann cells [32]. Here, we examined the in vivo impact of the absence of SCRIBBLE in murine skeletal muscle fibers. Although conditional *Scribble* knockout mice exhibited no changes in weight or viability, they demonstrated reduced force per weight, impaired neuromuscular transmission, and increased NMJ fragmentation. These muscles also transcribed less myosin heavy chain genes, and there was reduced interaction with proteins governing endocytic recycling.

We decided to monitor the involvement of SCRIBBLE in forming myotubes during development, their differentiation into myofibers, and their point of contact with nerve endings at the neuromuscular junctions. To this end, we examined the phenotype of the muscles of floxed *Scribble* mice. For this study, we selected mice that were 3–6 months old. Future studies should also investigate the influence of LAP proteins on old mice, which are known to naturally exhibit fragmented NMJs in late life. We induced the knockout using HSA-Cre recombinase-expressing mice. These HSA-Cre mice are known to initiate Cre recombinase expression in developing myotubes around E12, knocking out *Scribble* [36]. The role of LAP proteins in embryonic development, such as p.ex. prepatterning at NMJs was also not addressed in this study, but the prenatal role of SCRIBBLE has already been reported in an earlier study [15]. These muscle-specific *Scribble* knockout mice exhibit a mild phenotype, demonstrating reduced grip strength and impaired neuromuscular transmission (Figure 2B and Figure 6), suggesting that both skeletal muscles and neuromuscular junctions are affected in these knockout mice. In Muscle-specific *Scribble* knockout mice, mRNA levels of *Erbin* or *Lano* were not altered, but more LANO protein was detectable when determined by immunofluorescence staining. Future studies should possibly determine altered levels of ERBIN or LANO in muscle-specific *Scribble* knockout mice at different stages of development.

In this study, we examined transcript levels in the extensor digitorum longus (EDL) muscle, while protein levels were assessed in the GAS and primarily the SOL, representing two distinct muscle types. A brief pilot experiment found no detectable differences in transcript levels between EDL and SOL muscles for the markers analyzed). Furthermore, to date, there are no reports indicating a specific metabolic role for SCRIBBLE or other members of the LAP protein family. Our data, including COX staining (Figure 4), show no measurable differences between mutant and control SOL fibers, supporting the conclusion that global metabolic activity in this muscle is not altered. Although SOL was used for all other experiments, ensuring internal consistency, we recognize that the use of different muscle types for RNA and protein analyses presents a methodological limitation. This should be considered in future studies.

Consistent with compromised neuromuscular transmission in *Scribble* knockout mice, their NMJs are fragmented (Figure 7A–D). Quantal content, a measure of how much acetylcholine is released, did not differ between Scribble knockout mice and controls (Figure 6A). Conversely, treatment of diaphragms from Scribble knockout mice with d-tubocurarine led to a significant decrease in CMAP amplitudes in response to repetitive nerve stimuli (Figure 6B). For now, we speculate that neurotransmitter release is unaffected, but the safety factor is sustained. The safety factor is essentially a measure of the NMJ’s backup capacity to ensure reliable muscle contraction.

We look for further impairments in the skeletal muscles of *Scribble* knockout mice and found that the transcription of several myosin heavy chain genes was strongly diminished (Figure 5B). This is accompanied by a potentially related increase in the cross-sectional area of slow-type muscle fibers (Figure 5E,F). We acknowledge that CSA quantification based on myosin heavy chain immunostaining may result in less precise delineation of fiber borders compared with dystrophin or WGA staining. Nevertheless, all groups were analyzed under identical conditions, allowing valid relative comparisons. Minor variability in age or sex-related muscle characteristics cannot be entirely excluded, but is beyond the scope of our study.

To the best of our knowledge, SCRIBBLE has never been reported to directly regulate gene transcription. The detected change in myosin heavy chain gene expression may be indirect. It is notable that *Myh1* and *Myh2* transcript levels are reduced, but not *Myh4* (Figure 5B). Additionally, *Chrna* is reduced, but several other postsynaptic marker genes are not (Figure 7E). There were no further signs of muscle wasting or muscle hypertrophy in muscle-specific *Scribble* knockout mice.

At NMJs, CHRNs and MUSK were also shown to undergo endocytic sorting [53,54]. MUSK has been shown to undergo internalization via a clathrin- and dynamin-dependent pathway. There, it is transported to RAB7-positive endosomes for degradation. It is then recycled via RAB4- and RAB11-positive vesicles [55]. The trafficking of CHRNs can be studied using BTX, which binds to them selectively and irreversibly. When injected into live animals, BTX labels surface-exposed postsynaptic CHRNs, allowing the study of endo/lysosomal and recycling processes of CHRNs [56]. Imaging CHRNs labeled with BTX in the tibialis anterior muscles of live mice showed a steady state of approximately nine endocytic CHRN vesicles per NMJ, which increased drastically upon the overexpression of a GTPase-deficient, hyperactive Q79L mutant of RAB5 [56]. The involvement of LAP protein members ERBIN, LANO, and SCRIBBLE in endocytic recycling is investigated here. All three LAP proteins expressed in muscle cells interact with RAB5 (Figure 3D). Interestingly, the interaction between RAB5 and ERBIN or LANO is reduced in protein lysates from *Scribble* knockout muscles (Figure 3D,E), suggesting that SCRIBBLE acts as an upstream regulator of all LAP proteins involved in endocytic recycling in skeletal muscles. While our current data do not address downstream sorting and recycling routes, future studies should investigate whether SCRIBBLE influences PKA-, PKC-, CaMKII-, or myosin V-dependent recycling mechanisms at the postsynaptic membrane. Future studies should also consider the role of LAP proteins in endocytic recycling in different congenital myasthenic syndromes associated with CHRN mutations.

## Data Availability

All data generated or analyzed during this study are included in this published article and its Supplementary Materials.

## Supplementary Materials

The following supporting information can be downloaded at: https://www.mdpi.com/article/10.3390/cells14242005/s1, Table S1: Tabular presentation of oligonucleotide sequences.
