# Supplementary material for: The Cell Polarity Protein Scribble Is Involved in Maintaining the Structure of Neuromuscular Junctions, the Expression of Myosin Heavy Chain Genes, and Endocytic Recycling in Adult Skeletal Muscle Fibers
Source: Cells. 2025 Dec 16;14(24):2005. doi: 10.3390/cells14242005 (PMC12731447; doi:10.3390/cells14242005)
Supplement: Supplementary file 1 [file cells-14-02005-s001.zip › cells-3877372-supplementary.pdf]

Supplementary Table S1

Tabular presentation of oligonucleotide sequences

Genomic target	Orientation	Sequence
Chrna1 qPCR	forward	ACGCTGAGCATCTCTGTCTT
	reverse	TTGGACTCCTGGTCTGACTT
Chrng qPCR	forward	GGTCAATGTCAGCCTGAAGC
	reverse	GCACATGCATCCGTAACAGC
Densin 180 qPCR	forward	AGCACAGTCCACCACTCTTC
	reverse	AGTAGGTGCCTGAGGAGACA
Dok7 qPCR	forward	GAATTCGGTTCTCTGCTCAGTCTG
	reverse	CCAAGTCCATGTAGTGCAGCTG
ErbB2 qPCR	forward	GGCTCCGATGTGTTTGATGG
	reverse	AAGGGAGACTGAGGCCGAA
ErbB3 qPCR	forward	GAGCGGGGTGACGGGAGTAA
	reverse	GGGTCGCGAACAGTTCTCCC
ERBB2IP qPCR	forward	CAGGAGAGTGTTGCCAAGAT
	reverse	CATGGCCTTGTCTAGGAGAA
Erbin 71, 74 qPCR	forward	GCGTGTCTTCAACGGCTTCT
	reverse	ACTGTAGCCATTAGCCTGAA
Lrp4 qPCR	forward	CTGATGAACGCAACTGCACC
	reverse	CCTCGCACCATCTGACACTT
Lano qPCR	forward	GATCAGCGGCCTGACTTCTT
	reverse	CGGATGCAGAACATGGTGAG
Musk qPCR	forward	GCCTTGGTTGAAGAAGTAGC
	reverse	CTTGATCCAGGACACAGATG
Myh1 qPCR	forward	GGACCCACGGTCGAAGTTGCA
	reverse	GAACAGGCCCGAGTAGGTGTAGAT
Myh2 qPCR	forward	AGAGTCCCGAACGAGGCTGACTC
	reverse	TCAGGGTCGCTCCTGCTTCTGTT
Myh4 qPCR	forward	ATTTTCTGGGGGACAAGCTGCGG
	reverse	TGCTCCATGGCACCAGGAGTCT
Myod1 qPCR	forward	TGGCATGATGGATTACAGCGG
	reverse	GGTCTGGGTTCCCTGTTCTG
Myog qPCR	forward	CAGTACATTGAGCGCCTACA
	reverse	GCCTGACAGACAATCTCAGT
Pax7 qPCR	forward	GCTACCAGTACAGCCAGTATG
	reverse	GTCACTAAGCATGGGTAGATG
Rapsn qPCR	forward	CAAGACAAGGCTTTGGATGCC
	reverse	AGTGCCATTGTTTTGCAGGC
Rpl8 qPCR	forward	GTTCGTGTACTGCGGCAAGA
	reverse	ACAGGATTCATGGCCACACC
Scribble 6/12 qPCR	forward	GGGAGGCAACGACCTGGAAG
	reverse	ACAGCTCTCTGAGGGACTGCT
Scribble 30-33 qPCR	forward	AACTGCCAGCCAATGTGAAG
	reverse	ATGCGTAACCGTTCCTGATG
